# National Trends in the Use and Timing of Thoracic Endovascular Aneurysm Repair After Type B Aortic Dissection

**DOI:** 10.1016/j.atssr.2024.11.004

**Published:** 2024-11-15

**Authors:** Nicholas J. Goel, Siddharth Yarlagadda, Mikolaj Berezowski, Waseem Lutfi, Murat Yildiz, John DePaolo, Chase R. Brown, Wilson Y. Szeto, Nimesh D. Desai

**Affiliations:** 1 Division of Cardiovascular Surgery, Department of Surgery, University of Pennsylvania, Philadelphia, Pennsylvania; 2 Leonard Davis Institute of Health Economics, University of Pennsylvania, Philadelphia, Pennsylvania

## Abstract

**Background:**

The optimal management of uncomplicated type B aortic dissection (TBAD) has become controversial in recent years, especially concerning the use and timing of thoracic endovascular aneurysm repair (TEVAR). Here, we analyze a large national cohort over 11 years to understand the current national landscape of TBAD management, trends over time, and disparities in care across the United States.

**Methods:**

Admissions for acute TBAD from 2010 to 2020 were identified in the Nationwide Readmissions Database, a large nationally representative sample of hospital admissions across 30 states. Patients were tracked through the calendar year to understand midterm treatment strategies after TBAD, specifically the use and timing of TEVAR.

**Results:**

Overall, 10,628 patients with acute TBAD were identified, of whom 7483 (70.4%) were discharged alive after upfront medical management. Among medically managed TBAD patients, 8.8% underwent interval TEVAR by 300 days. In addition to age and comorbidity burden, residence in a low-income ZIP Code (odds ratio, 0.75; 95% CI, 0.61-0.91; *P* = .004) and treatment at a teaching hospital (odds ratio, 1.29; 95% CI, 1.01-1.66; *P* = .042) were independently associated with the likelihood of interval TEVAR. Among all TBADs treated with TEVAR nationally from 2010 to 2019, a clear year-by-year trend toward greater use of TEVAR in the postacute period was observed (*P* = .004). Among all TEVARs for TBAD nationally, 40% were performed >14 days after dissection in 2019 compared with 17% in 2010.

**Conclusions:**

Nationally, interval TEVAR after medically managed TBAD has become much more common, although important disparities remain in its use.

## Visual Abstract

**Figure:**
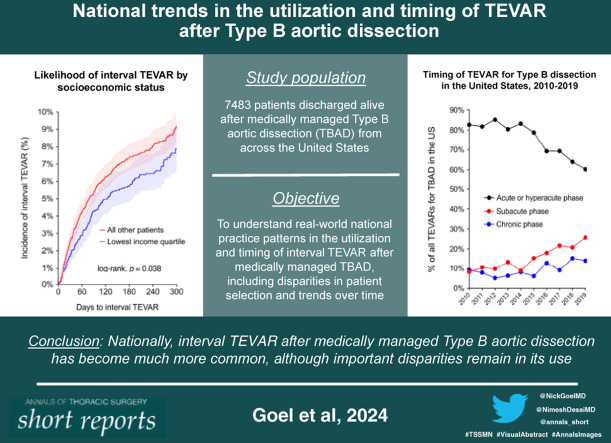

**In Short**

▪ Today ∼10% of patients with medically managed type B aortic dissection (TBAD) undergo interval thoracic endovascular aneurysm repair (TEVAR) within 300 days of their dissection, and use of interval TEVAR after medically managed TBAD has become far more common nationally in the last decade.
▪ Median time to interval TEVAR after medically managed TBAD is ∼90 days for those receiving TEVAR electively and 35 days for patients who require unplanned TEVAR.
▪ Very important disparities remain in the use of interval TEVAR after medically managed TBAD.

Despite recent interest and attention in the role of thoracic endovascular aneurysm repair (TEVAR) in type B aortic dissection (TBAD) management, studies characterizing the real-world national landscape of TEVAR used for TBAD are lacking. As a result, the effect of new evidence and shifting guidelines on current national practice in this field remains unclear. Furthermore, significant disparities are known to exist in the care of patients with aortic disease and that such disparities are often most prominent in the application of new techniques, technology, and evidence.^1^^,^^2^ We believe that a close investigation of real-world practice patterns is key to understanding outcomes, guiding future research, and ensuring optimal and equitable care for all patients with TBAD. Thus, the current study describes a large, nationally representative cohort of TBAD patients over 11 years and analyzes outcomes, management strategies, and trends over time.

## Patients and Methods

Data for this study were derived from the Nationwide Readmissions Database (NRD). The NRD is a large, public, all-payer database of inpatient hospital encounters sponsored by the Healthcare Cost and Utilization Project.^3^ By design, the NRD is nationally representative—incorporating data from 30 different states accounting for 61% of the total United States resident population. The NRD uses patient linkage numbers to track patients across all admissions to all hospitals in a given state through a calendar year. Further details on the NRD are available online.^3^ The study meets the University of Pennsylvania Institutional Review Board criteria for exemption from review according to 45 CFR 46.104, category 4.

**Supplemental Figure 1:**
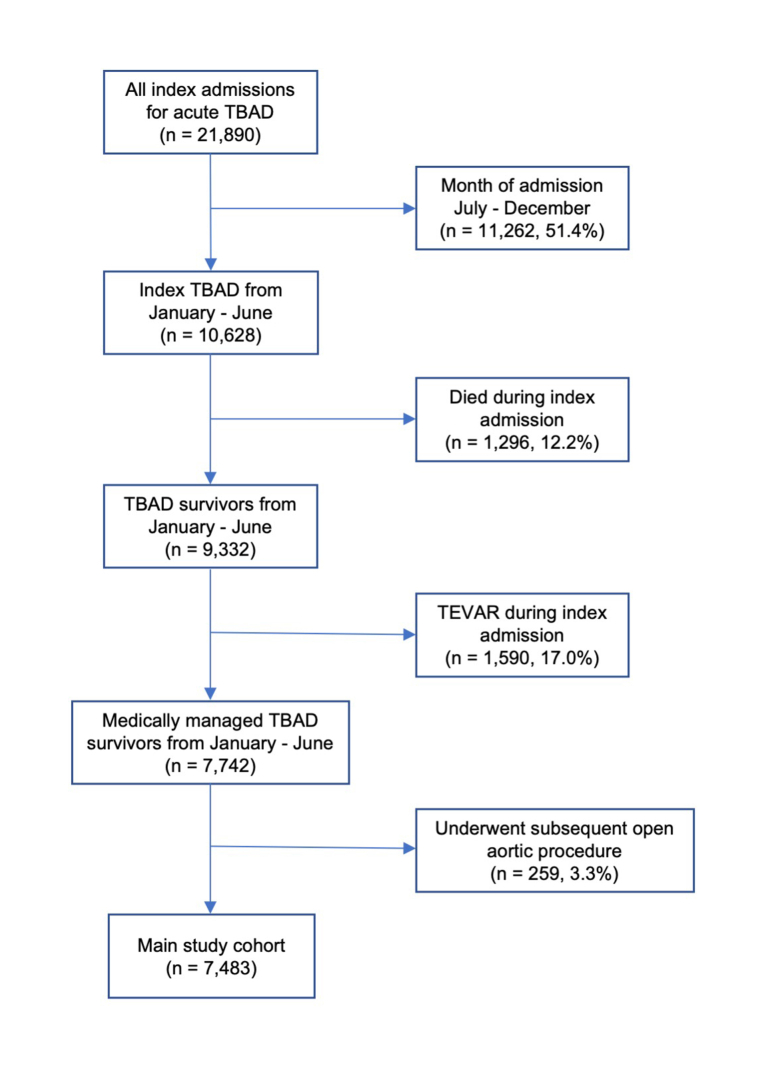


The study population included patients admitted for acute TBAD from 2010 to 2020. TBAD was identified by *International Classification of Diseases* 9th and 10th revision (ICD-9 and ICD-10) diagnosis codes. Medically managed TBAD was defined as TBAD wherein a patient was discharged alive from index hospitalization without having undergone TEVAR or open surgery. Because the NRD only tracks individual patients through a single calendar year, only index admissions in January through June were considered in this study. Doing this ensured a minimum of 167 days of follow-up for every patient included in the main study cohort and a maximum follow-up of 334 days. Construction of the main study cohort is illustrated in Supplemental Figure 1.

Univariate comparisons among categorical variables were made by χ^2^ tests. Kaplan-Meier curves were compared using the log-rank test. For tests of trends of over time, we used the nonparametric Mann-Kendall test. Statistical analyses were performed using Stata 15 (StataCorp LP, College Station, TX) and GraphPad Prism 10 (GraphPad Software Inc, La Jolla, CA) statistical software.

## Results

**Supplemental Figure 2:**
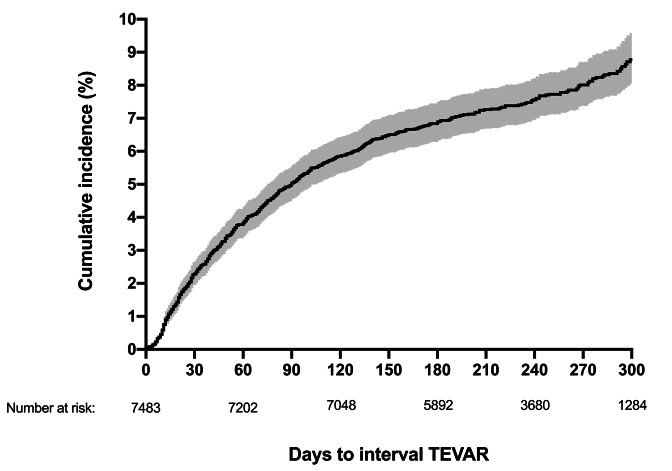


We identified 21,890 index admissions for acute TBAD from the NRD from 2010 to 2020. Of these, 7483 were patients with medically managed TBAD from the first half of the calendar year and thus eligible for the main study cohort (Supplemental Figure 1). Of all acute TBAD patients considered, the rate of in-hospital mortality was 12.2% (Supplemental Figure 2). Among patients who survived TBAD admission, 17.0% underwent TEVAR during the index admission. The remaining 83.0% of survivors were considered medically managed TBAD; that is, those patients undergoing a strategy of upfront nonsurgical management.

**Figure 1:**
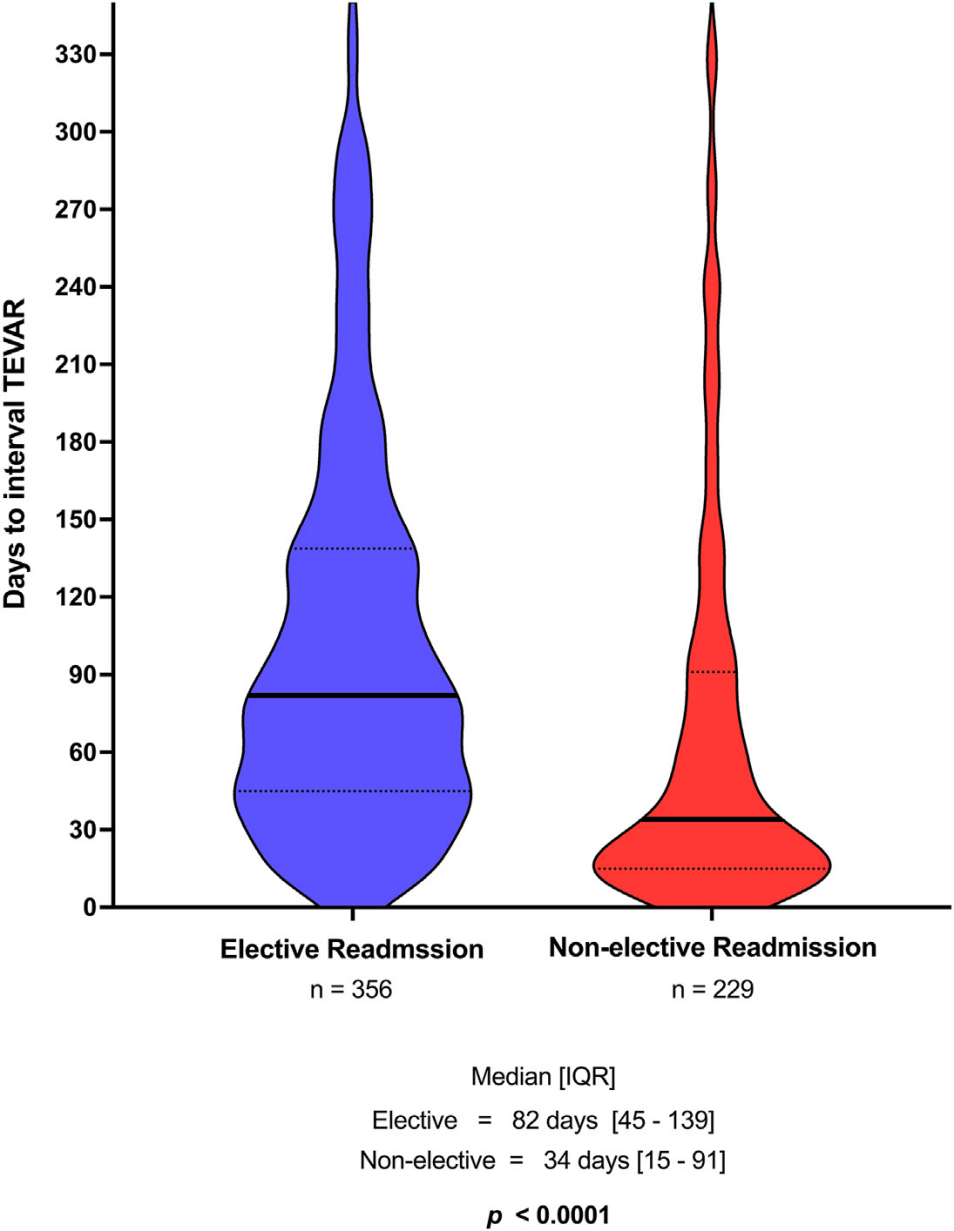
Violin plot demonstrates the timing of interval thoracic endovascular aneurysm repair (TEVAR) after medically managed type B aortic dissection during elective vs unplanned readmissions. (IQR, interquartile range.)

**Supplemental Figure 3:**
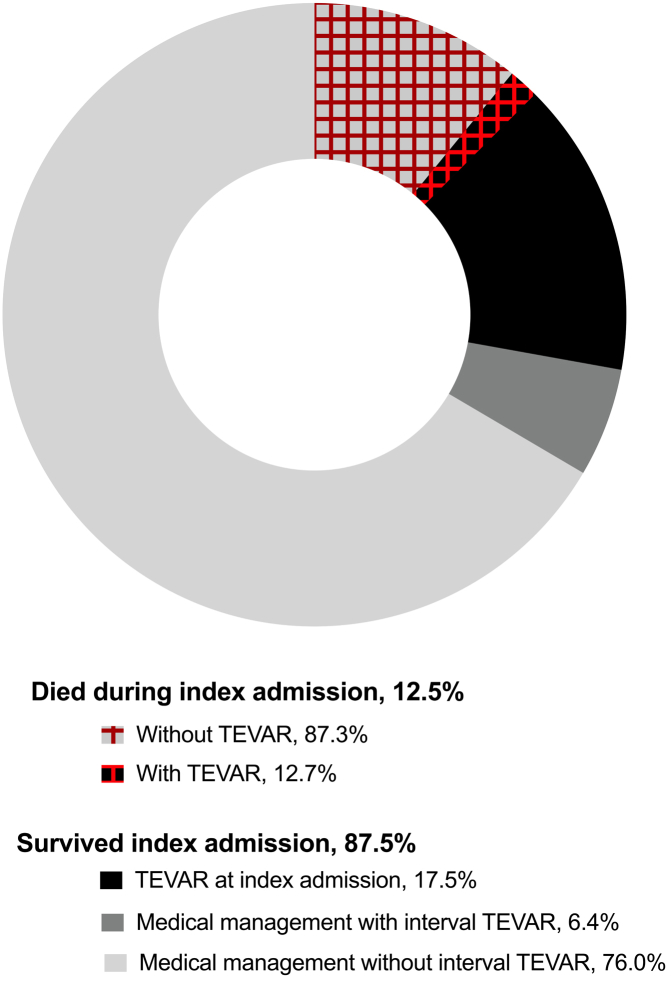


The median follow-up period for medically managed TBAD patients was 228 days, and minimum follow-up was 167 days. Through 300 days, the cumulative incidence of interval TEVAR among medically managed TBAD patients was 8.8% (95% CI, 8.0%-9.6%) (Supplemental Figure 3). Timing of interval TEVAR after medically managed TBAD is described in Figure 1. For medically managed TBAD patients undergoing interval TEVAR in the follow-up period during an elective readmission, the median time from initial TBAD presentation to TEVAR was 82 days (interquartile range, 45-139 days). For interval TEVAR during nonelective readmission, the median time to TEVAR was 34 days (interquartile range, 15-91 days). Thus, nonelective interval TEVARs occurred much sooner than elective interval TEVARs (*P* < .0001). Of interval TEVARs in the follow-up period, 61% were elective and 39% were nonelective.

**Figure 2:**
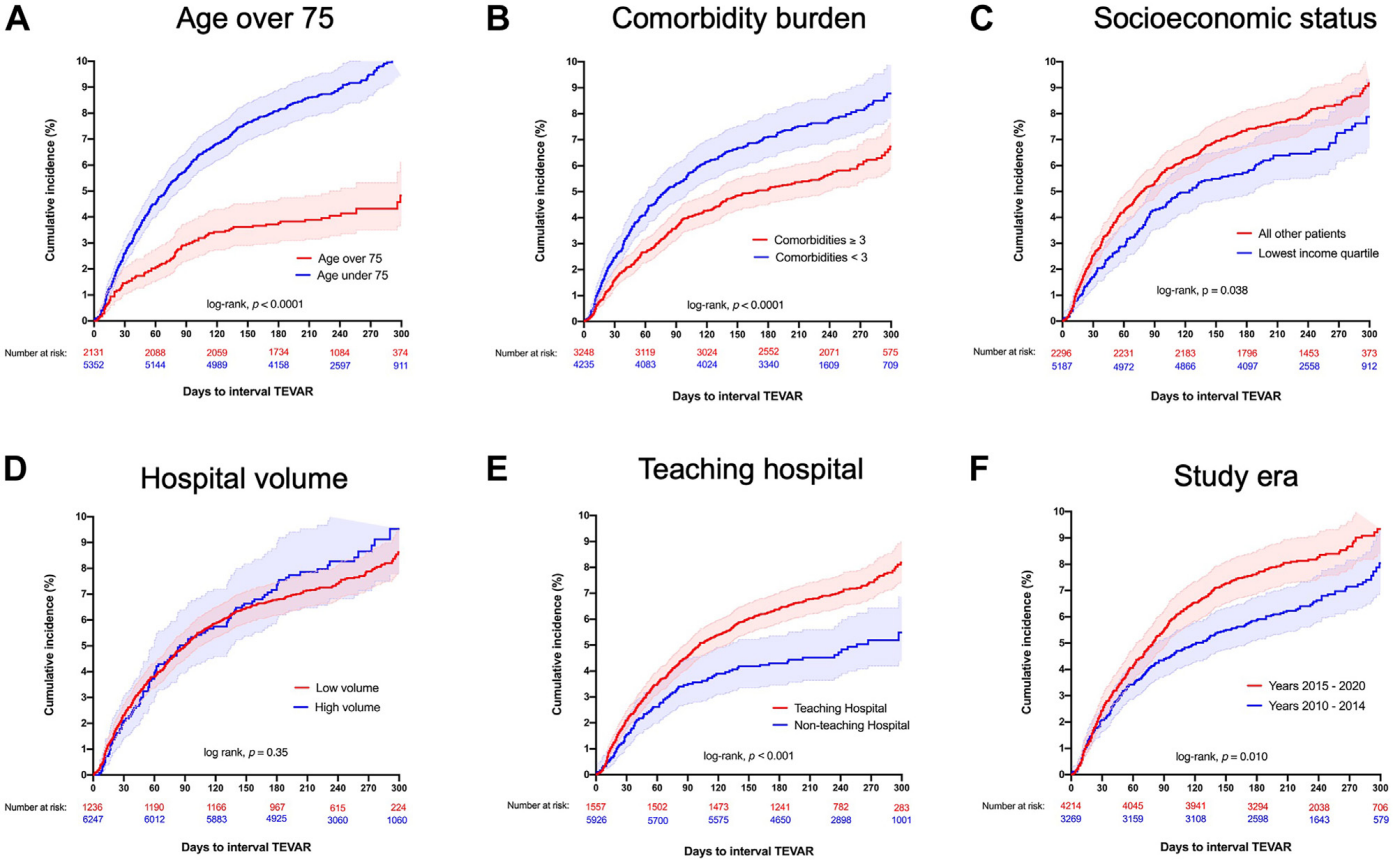
Cumulative incidence of interval thoracic endovascular aneurysm repair (TEVAR) in patients who have undergone upfront medical management of an acute type B aortic dissection stratified by key factors of (A) age >75 years, (B) comorbidity burden, (C) socioeconomic status, (D) hospital volume, (E) treatment at a teaching hospital, and (F) study era. The shaded areas indicate the 95% CI.

A comparison of medically managed TBAD patients who did and did not undergo interval TEVAR during the follow-up period is given in the Table. Univariate and multivariate analysis considered 15 patient- and hospital-level covariates in relation to the likelihood of interval TEVAR after medically managed TBAD. Of the 15 factors considered, age showed the strongest independent association with interval TEVAR by far. At 300 days after medically managed TBAD, the cumulative incidence of interval TEVAR was 4.8% (95% CI, 3.8%-6.1%) in patients aged >75 years and 10.4% (95% CI, 9.4%-11.4%) in patients <75 years (log-rank, *P* < .0001) (Figure 2A).

**Table:** Patient and Hospital Factors Associated With Interval Thoracic Endovascular Aneurysm Repair After Medically Managed Type B Aortic Dissection

Variable	No Interval TEVAR	Interval TEVAR	Total	Univariate Analysis		Multivariable Analysis	
	(n = 6898)	(n = 585)	(N = 7483)	Odds Ratio (95% CI)	*P* Value	Odds Ratio (95% CI)	*P* Value
Demographics							
Age >75 years	2040 (29.6)	91 (15.6)	2131 (28.5)	0.44 (0.34-0.55)	**<.0001**	0.45 (0.35-0.57)	**<.0001**
Male sex	4142 (60.1)	342 (58.5)	4484 (59.9)	0.94 (0.79-1.12)	.28	0.85 (0.71-1.01)	.07
Lowest income quartile	2007 (29.7)	145 (25.2)	2152 (29.3)	0.80 (0.65-0.98)	**.024**	0.75 (0.61-0.91)	**.004**
Comorbidities							
Hypertension	5723 (82.0)	511 (87.4)	6234 (83.3)	1.42 (1.10-1.85)	**.006**	1.52 (1.17-1.96)	**.002**
Peripheral arterial disease	3575 (51.8)	302 (51.6)	3877 (51.8)	0.99 (0.83-1.18)	.92	0.74 (0.58-0.94)	**.015**
Chronic kidney disease	1418 (20.6)	96 (16.4)	1514 (20.2)	0.76 (0.60-0.95)	**.017**	0.84 (0.66-1.06)	.14
Obesity	1021 (14.8)	91 (15.6)	1112 (14.9)	1.06 (0.89-1.34)	.62	0.97 (0.76-1.24)	.82
Atrial fibrillation or flutter	1121 (16.3)	58 (9.9)	1179 (15.8)	0.57 (0.42-0.75)	**<.001**	0.67 (0.50-0.90)	**.008**
Diabetes	1148 (16.6)	86 (14.7)	1234 (16.5)	0.86 (0.67-1.10)	.22	0.87 (0.68-1.11)	.27
Smoking	2433 (35.3)	210 (35.9)	2643 (35.3)	1.02 (0.86-1.23)	.76	0.99 (0.83-1.20)	.95
Congestive heart failure	1109 (16.1)	60 (10.3)	1169 (15.6)	0.60 (0.45-0.79)	**<.001**	0.66 (0.50-0.89)	**.006**
Chronic liver disease	235 (3.4)	18 (3.1)	253 (3.4)	0.90 (0.52-1.46)	.67	0.90 (0.55-1.47)	.67
Hospital factors							
Teaching hospital	5499 (79.7)	496 (84.8)	5995 (80.1)	1.41 (1.12-1.81)	**.003**	1.29 (1.01-1.66)	**.042**
High volume for TBAD	1132 (16.4)	104 (17.7)	1236 (16.5)	1.10 (0.87-1.38)	.89	0.99 (0.79-1.26)	.96
Era				1.25 (1.05-1.50)	**.011**	1.45 (1.14-1.86)	**.003**
2010-2014	3042 (44.1)	226 (38.6)	3268 (43.7)				
2015-2020	3856 (55.9)	359 (61.4)	4215 (56.3)				

Data are presented as n (%). Bold *P* values are statistically significant (*P* < .05).

TBAD, type B aortic dissection; TEVAR, thoracic endovascular aortic repair.

Socioeconomic status (SES) also demonstrated a strong independent association with treatment strategy on multivariable analysis (Table). Patients living in ZIP Codes in the lowest quartile of median household income were less likely to undergo interval TEVAR after medically managed TBAD (odds ratio, 0.75; 95% CI, 0.61-0.91; *P* = .004). This effect was independent of the other factors considered in the study, including whether a patient was cared for at a high-volume center or a teaching hospital. The effect of SES on treatment strategy was further demonstrated in Figure 2C, which noted a significantly lower cumulative incidence of interval TEVAR in patients of lower SES (log-rank, *P* = .038).

**Figure 3:**
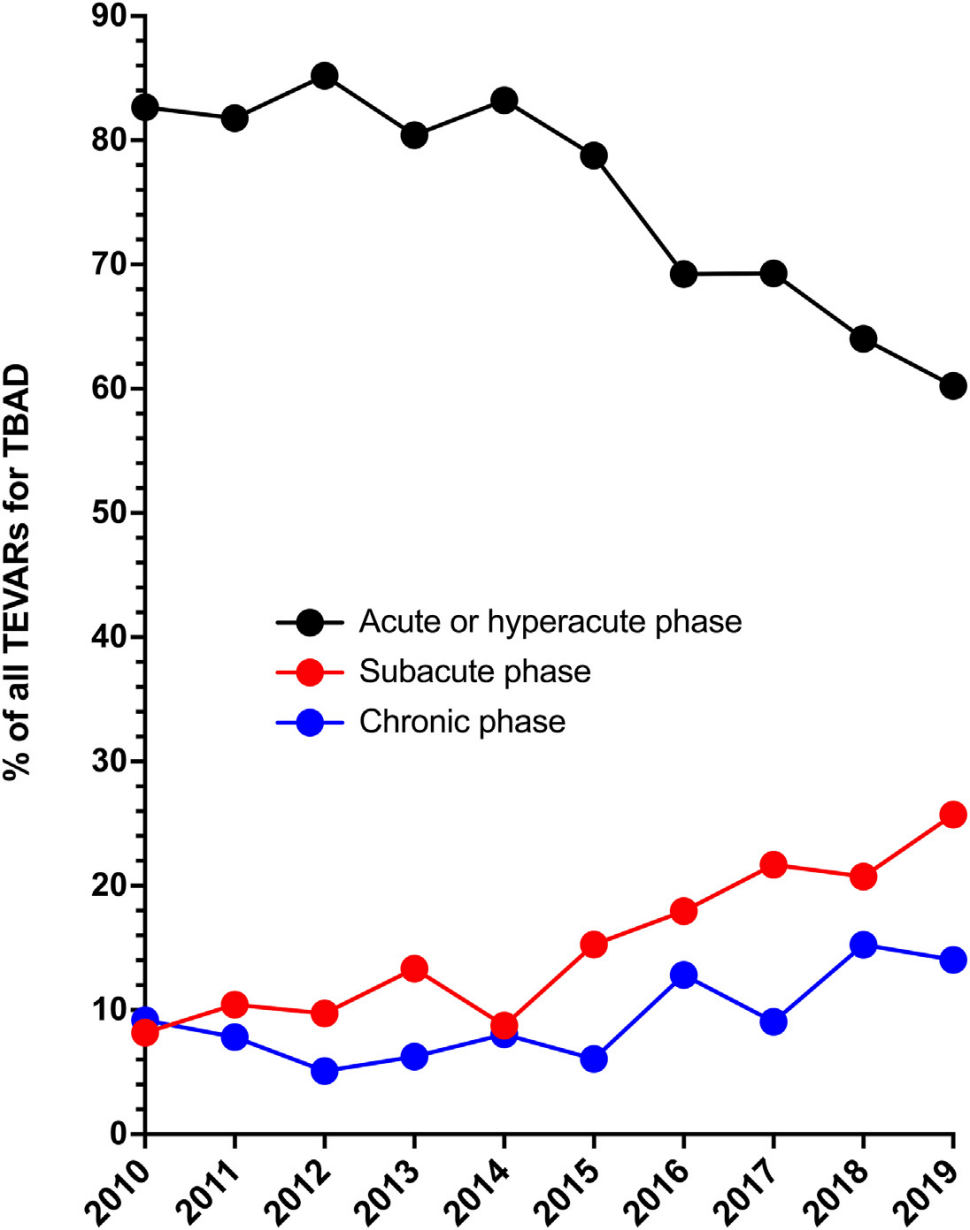
National trend in the timing of all thoracic endovascular aortic repairs (TEVARs) performed for type B aortic dissection (TBAD) in the United States from 2010 to 2019.

In this study, we also observed that the treatment strategy for medically managed TBAD changed over time, with interval TEVAR becoming increasingly common in the later part of the 2010 to 2020 decade. Figure 3 demonstrates the year-over-year trend in the timing of all TEVARs performed at any point during the follow-up period in all patients who presented with acute TBAD from 2010 to 2019 included in the study. Comparing 2010 with 2019, 82.7% vs 60.2% of TEVARs for TBAD occurred in the hyperacute or acute phase (≤14 days of the initial presentation), 8.2% vs 25.7% occurred in the subacute phase (15-90 days), and 9.2% vs 14.0% in the chronic phase (>90 days), respectively. The Mann-Kendall test for trend was strongly significant for an increasing proportion of postacute TEVAR over time (*P* = .004). This strongly suggests greater use of interval TEVAR after medically managed TBAD over time, consistent with the result shown in Figure 2F.

## Comment

The present study analyzes national trends in the use and timing of interval TEVAR in a cohort of nearly 7500 medically managed TBADs across the United States. This study describes postdischarge treatment strategies after medically managed TBAD in a very large, nationally representative cohort. Before this study, data on the postdischarge use of different treatment strategies in this population were limited to much smaller cohort studies from academic centers or studies of the Medicare-only patient population, not designed to represent the national experience.^4^^,^^5^ This knowledge gap persisted because collecting data on large groups of patients longitudinally across multiple discrete hospital admissions is inherently challenging.

The NRD was specifically designed to overcome these challenges. The NRD is nationally representative and samples millions of patients of all payers from 30 different states through an entire calendar year. Thus, the NRD is particularly well-suited for understanding treatment strategies after medically managed TBAD across the whole of the United States population.

This study identified a substantial change in national practice patterns from 2010 to 2020, with a trend toward greater use of interval TEVAR after medically managed TBAD and a shift in the timing of TEVARs after TBAD away from the acute setting. Indeed, over just 10 years, the proportion of all TEVARs for TBAD performed after the acute period more than doubled, from 17% in 2010 to 40% in 2019. This major in shift in national practice likely reflects the growing body of evidence supporting early interval TEVAR for uncomplicated medically managed TBAD.^6^^,^^7^ A major paradigm shift in the field of aortic surgery is well underway, as demonstrated by the results of this study. This study also identified a difference in treatment strategy according to a patient’s SES, wherein lower SES patients were less likely to receive interval TEVAR. This may reflect the difficulty these patients face in maintaining close follow-up—an important factor for clinicians to consider.

### Limitations

This study has important limitations to note. As with any study based on claims data from a large multi-institutional data set, granularity can be limited. Another limitation is the inability to account for out-of-hospital mortality. On time-to-event analysis for interval TEVAR, mortality represents an important competing risk. However, because the NRD does not measure out-of-hospital mortality, the competing risk of mortality is not completely accounted for in this study.

### Conclusions

The present study characterizes the current national landscape with respect to postdischarge treatment strategies for patients with TBADs. In the United States, ∼10% of patients will undergo an interval TEVAR in 300 days after a medically managed TBAD, and the prevalence of interval TEVAR has increased substantially since 2010, reflecting a paradigm shift in the field toward greater use of interval TEVAR. Patients who do undergo a planned interval TEVAR after medically managed TBAD do so an average of ∼80 days after dissection. Importantly, significant disparities are evident in how medically managed TBAD patients are treated after discharge, including disparities according to SES.

## References

[1] Newell P., Zogg C., Asokan S. (2024). Race and socioeconomic disparities in proximal aortic surgery. Ann Thorac Surg.

[2] Kabbani L.S., Wasilenko S., Nypaver T.J. (2016). Socioeconomic disparities affect survival after aortic dissection. J Vasc Surg.

[3] Agency for Healthcare Research and Quality Overview of the Nationwide Readmissions Database (NRD). https://hcup-us.ahrq.gov/nrdoverview.jsp.

[4] Torrent D.J., McFarland G.E., Wang G. (2021). Timing of thoracic endovascular aortic repair for uncomplicated acute type B aortic dissection and the association with complications. J Vasc Surg.

[5] Mody P.S., Wang Y., Geirsson A. (2014). Trends in aortic dissection hospitalizations, interventions, and outcomes among Medicare beneficiaries in the United States, 2000-2011. Circ Cardiovasc Qual Outcomes.

[6] Nienaber C.A., Kische S., Rousseau H. (2023). Endovascular repair of type B aortic dissection: long-term results of the randomized investigation of stent grafts in aortic dissection trial. Circ Cardiovasc Interv.

[7] VIRTUE Registry Investigators (2014). Mid-term outcomes and aortic remodeling after thoracic endovascular repair for acute, subacute, and chronic aortic dissection: the VIRTUE Registry. Eur J Vasc Endovasc Surg.

