# Supplementary material for: National Trends in the Use and Timing of Thoracic Endovascular Aneurysm Repair After Type B Aortic Dissection
Source: Ann Thorac Surg Short Rep. 2024 Nov 15;3(2):379–84. doi: 10.1016/j.atssr.2024.11.004 (PMC12167546; doi:10.1016/j.atssr.2024.11.004)
Supplement: Supplemental Material [file mmc1.docx]

**Supplemental Figure captions**

**Supplementary Figure 1.** Diagram illustrating cohort construction

**Supplementary Figure 2.** Early treatment strategies among patients with acute TBAD in a national cohort

**Supplementary Figure 3.** Cumulative incidence of interval TEVAR after upfront medical management of acute TBAD
